# Immune cells with senescence-related transcriptional signatures orchestrate the inflammatory continuum in osteoarthritis synovium: a single-cell and machine learning study

**DOI:** 10.3389/fimmu.2026.1774722

**Published:** 2026-04-17

**Authors:** Chunrong He, Xianghan Wang, Peiguan Huang, Jianxin Jiang, Xiaoxu Wang

**Affiliations:** 1 Department of Trauma Medical Center, Daping Hospital, State Key Laboratory of Trauma and Chemical Poisoning, Army Medical University, Chongqing, China; 2 Department of Orthopedic Surgery, The Second Affiliated Hospital, University of South China, Hengyang, Hunan, China

**Keywords:** immune microenvironment, immunosenescence, machine learning, osteoarthritis, RHOB, synovitis

## Abstract

**Background:**

Synovitis is a key driver of osteoarthritis (OA), characterized by a chronic inflammatory microenvironment. However, the contribution of immune-cell senescence to synovial pathology and its therapeutic potential remains poorly understood. This study aims to map the senescent immune landscape and identify actionable targets for OA therapy.

**Methods:**

Single-cell RNA dataset (GSE152805) of OA synovium was used to identify immune subsets with senescence-related transcriptional signatures and subset-specific gene signatures. These gene signatures served as machine-learning features to train and validate diagnostic models in a combine dataset of 4 OA transcriptomic datasets (GSE55235, GSE55457, GSE82107, GSE169077) using Random Forest, Support Vector Machine, Gradient Boosting Machine, and eXtreme Gradient Boosting. Immunofluorescence staining and Western blotting of human synovial tissues validated macrophage-associated markers. Pseudotime trajectory and cell–cell communication analyses defined temporal dynamics and intercellular communication. Drug–gene interaction analysis and molecular docking were performed to assess therapeutic potential.

**Results:**

Macrophages, mast cells, and dendritic cells were identified as the immune populations with senescence-related transcriptional signatures. Pseudotime and communication analyses revealed a coordinated inflammatory continuum: macrophages initiated and sustained synovial inflammation, mast cells amplified intermediate inflammatory responses, and dendritic cells contributed to late-stage matrix remodeling. Machine-learning models identified hub biomarkers, with RHOB and PDK4 emerging as the macrophage-associated biomarkers with therapeutic potential. RHOB and PDK4 were markedly upregulated and colocalized with CD68^+^ macrophages in OA synovium. Drug prediction suggested CHEMBL1797159 as a potential ligand for RHOB and dichloroacetate as a repurposable modulator of PDK4.

**Conclusion:**

This study characterizes the transcriptomic landscape of senescence-related features in the OA synovial microenvironment. We identify RHOB and PDK4 as key macrophage-associated therapeutic targets, providing novel insights for immunomodulatory strategies in OA treatment.

## Introduction

Osteoarthritis (OA) is the most prevalent chronic joint disease mainly affecting the elderly population, characterized by chronic intra-articular inflammation and progressive degeneration of articular cartilage (1). Globally, OA affects more than 500 million people, with a prevalence exceeding 50% among individuals aged 65 years and older (2). Yet effective disease-modifying therapies for OA remain unavailable. Accumulating evidence highlights synovitis as a central driver of OA progression, closely linked to joint pain, inflammation, and structural remodeling (3, 4). Within the synovial immune microenvironment, macrophages, mast cells, and dendritic cells orchestrate cytokine secretion, inflammation responses, and matrix remodeling (5–7).

Cellular senescence has emerged as a key pathogenic mechanism in age-related diseases and is characterized by permanent cell-cycle arrest, metabolic and transcriptional reprogramming, and production of a senescence-associated secretory phenotype (SASP) (8). OA, as an aging-related disease, is associated with accumulation of senescent chondrocytes and synovial cells contributing to cartilage degradation and intra-articular inflammation (9–11). However, despite the central role of synovial inflammation in OA, the senescence features of immune cells within the synovium remain poorly defined. It is unclear which immune subsets preferentially acquire senescent phenotypes, how senescence reshapes their functions, and whether senescence-associated biomarkers exist that could serve as therapeutic targets. Addressing these gaps is essential for understanding how immunosenescence shapes OA progression.

Single-cell RNA sequencing (scRNA-seq) provides high-resolution dissection of synovial immune heterogeneity, enabling precise identification of immune subsets and quantification of senescence signatures at the single-cell level (12). Although previous studies have mapped synovial cellular lineages and inflammatory signaling networks (13, 14), few have explored immune-cell senescence or senescence-associated biomarkers. Moreover, senescent immune cells may interact with each other to modulate synovial immune microenvironment. Pseudotime trajectory and intercellular communication analyses can reconstruct these transitions and signaling interactions in silico, offering mechanistic insight into how senescent immune populations may evolve and influence each other (15, 16).

Machine-learning methods have become powerful tools for disease classification and biomarker discovery, owing to their ability to capture complex nonlinear patterns (17). Four machine-learning algorithms applied in this study—Random Forest (RF), Support Vector Machine (SVM), Gradient Boosting Machine (GBM), and eXtreme Gradient Boosting (XGBoost)—allow cross-validation of feature importance across different modelling principles. Features consistently prioritized across multiple algorithms therefore carry greater robustness and biological plausibility than those derived from a single method. Leveraging immune-cell-specific transcriptional signatures derived from scRNA-seq further improves model specificity and facilitates identification of candidate therapeutic targets associated with senescent immune subsets.

In this study, we aimed to define senescent immune subsets in OA synovium and identify immune-cell-associated biomarkers with therapeutic potential. We integrated single-cell analysis, multi-algorithm machine-learning modelling, clinical validation, drug discovery, pseudotime trajectory and intercellular communication analyses to uncover therapeutic targets within the senescent synovial immune microenvironment.

## Methods

### Synovial tissue collection

Fresh synovial tissues were collected from patients undergoing knee surgery at the Second Affiliated Hospital of University of South China. All procedures were approved by the Clinical Ethics Committee of The Second Affiliated Hospital, University of South China (Approval No.: 2025100). The OA group included patients older than 65 years who underwent total knee arthroplasty for end-stage OA (n = 5), and the control group included patients younger than 30 years undergoing arthroscopic surgery for acute knee injury (n = 5).

### Immunofluorescence staining

Immediately after excision, tissues were embedded in optimal cutting temperature (OCT) compound, snap-frozen in liquid nitrogen, and stored at −80 °C until sectioning. Frozen sections (4 μm) were cut using a cryostat and mounted on glass slides. Sections were fixed with 4% paraformaldehyde for 15 min, permeabilized with 0.2% Triton X-100 for 10 min, and blocked with 10% normal goat serum for 1 h at room temperature. Immunofluorescence staining was then performed overnight at 4 °C using primary antibodies purchased from Proteintech, including PDK4 (polyclonal, Cat. No. 12949-1-AP), RHOB (polyclonal, Cat. No. 14326-1-AP), and CD68 (monoclonal, Cat. No. 66231-2-lg). After PBS washing, sections were incubated with secondary antibodies (conjugated with FITC and Coralite594, 1:500 dilution) (Cat No.SA00013-4 and Cat No. SA00003-1) for 1 h at room temperature in the dark. Nuclei were counterstained with DAPI and mounted with anti-fade reagent.

Fluorescence images were acquired using a Leica confocal laser-scanning microscope under identical settings for all samples. Quantitative and colocalization analyses were performed using Fiji ImageJ software.

### Western blotting

Total protein was extracted from synovial tissues of OA patients and controls using RIPA lysis buffer supplemented with protease inhibitors. Protein concentrations were determined using a bicinchoninic acid (BCA) protein assay kit (Pierce, USA). Equal amounts of protein were separated by 10% SDS–PAGE and transferred onto polyvinylidene fluoride (PVDF) membranes. Prestained Protein Marker X (10–180 kDa; Servicebio, China) was used to estimate molecular weight. Membranes were blocked with Tris-buffered saline containing 0.1% Tween 20 (TBST) supplemented with 5% skim milk at room temperature for 1 h, followed by incubation with primary antibodies against RHOB (ABclonal, Cat. No. A22258), PDK4 (ABclonal, Cat. No. A13337), β-Tubulin (Santa Cruz Biotechnology, Cat. No. sc-5274), or GAPDH (Proteintech, Cat. No. 60004-1-Ig) at 4 °C overnight. After washing with TBST, membranes were incubated with appropriate HRP-conjugated secondary antibodies at room temperature for 2 h. Protein bands were visualized using enhanced chemiluminescence (ECL) reagents (Millipore) and captured with a chemiluminescence imaging system. Band intensities were analyzed using ImageJ software.

### Data sources and preprocessing

Bulk RNA-seq datasets of knee OA synovium and cartilage were obtained from GEO via GEOquery (GSE55235, GSE55457, GSE82107, GSE169077) (**Table 1**) (18–20). In total, a combined dataset comprised 36 OA and 32 non-OA samples (GSE55235: 10 OA/10 control; GSE55457: 10/10; GSE82107: 10/7; GSE169077: 6/5). Batch effects across studies were mitigated with the “ComBat” algorithm in the “sva” R package (21). To confirm successful harmonization, we performed principal-component analysis (PCA) on expression matrices before and after correction. Single-cell RNA-seq dataset (GSE152805) from OA synovium were obtained for validation (14).

**Table 1:** GEO microarray chip information.

Dataset	GSE55235	GSE55457	GSE82107	GSE169077	GSE152805
Platform	GPL96	GPL96	GPL570	GPL96	GPL20301
Species	Homo sapiens	Homo sapiens	Homo sapiens	Homo sapiens	Homo sapiens
Tissue	Osteoarthritis synovial membrane	Osteoarthritis synovial membrane	Osteoarthritis synovial membrane	knee cartilage	Osteoarthritis synovial membrane
Samples in OA Group	10	10	10	6	3
Samples in Control Group	10	10	7	5	
Reference	PMID: 24690414	PMID: 24690414	PMID: 27870898	NA	PMID: 32616761

### Senescence scoring of synovial cells

A list of 279 senescence-associated genes (**Supplementary Table 2**) from previous literature was utilized to quantify the degree of cellular senescence at the single-cell level (22, 23). Two approaches were applied to score senescence enrichment. First, using “AddModuleScore” function of “Seurat”, each cell in the GSE152805 dataset was assigned a senescence module score based on the averaged expression of the 279 senescence-related genes relative to aggregated control features. Second, the “AUCell” R package was employed to compute an area under the curve (AUC) score for each cell, which reflects the proportion of the senescence signature genes among the cell’s highest-expressed transcripts. Cells with higher scores were classified as senescence-enriched subpopulations.

### Analysis of single-cell RNA-seq dataset

Raw count data from the single-cell RNA-seq dataset GSE152805 (OA synovial tissue) were processed using the “Seurat” R package (21). Cells expressing fewer than 200 genes, or with a mitochondrial transcript fraction exceeding 20%, were excluded. Genes detected in fewer than 3 cells were removed. After log-normalization (“LogNormalize”) and scaling (“ScaleData”), 2,000 highly variable genes were identified using the “FindVariableFeatures” function with the “vst” approach. Principal component analysis (PCA) was then performed, and the top 20 principal components (PCs) were selected for downstream analysis based on “ElbowPlot” inspection. Using these PCs, cell clustering was conducted with “FindNeighbors” and “FindClusters” (resolution = 0.25), and the resulting transcriptional landscape was visualized via Uniform Manifold Approximation and Projection (UMAP). Cell-type annotation was performed using “SingleR” with the HumanPrimaryCellAtlasData reference (24), and the assignments were manually refined based on canonical marker expression. Marker expression patterns across cell clusters were visualized using “DotPlot”.

Cluster-specific marker genes were identified using “FindAllMarkers”, which applies the Wilcoxon rank-sum test to compare the expression of each gene in a given cluster against all remaining cells. Genes were ranked according to fold change and statistical significance, and those meeting the criteria of |log_2_FC| > 1 and adjusted p-value < 0.05 were considered single-cell differentially expressed genes (scDEGs), which were identified from the synovial single-cell dataset (GSE152805). These scDEG sets represent the distinct molecular features of each cell population and form the foundation for downstream analyses, including the construction of machine learning–based diagnostic models. In particular, the characteristic gene signatures of monocyte/macrophages, mast cells, and dendritic cells were extracted and summarized in **Supplementary Table 1**.

### Functional enrichment analysis

Functional enrichment of immune cell scDEGs was carried out with R package “clusterProfiler” (25). We performed Gene Ontology (GO) enrichment analysis and the Kyoto Encyclopedia of Genes and Genomes (KEGG) pathway enrichment analysis. To quantify the enrichment of immune cell–specific scDEGs in bulk transcriptomic datasets, Gene Set Variation Analysis (GSVA) was performed using the R package “GSVA” (26). For each dataset (GSE55235, GSE55457, GSE82107, and GSE169077), GSVA scores were calculated for every sample to represent the relative expression level of these immune cell gene signatures.

### Machine-learning–based diagnostic modelling

To identify potential key biomarkers of OA synovium immune cells, we developed transcriptome-based machine-learning models using immune cell–specific scDEGs as predictive features. Four bulk transcriptome datasets (GSE55235, GSE55457, GSE82107, and GSE169077) were integrated to create a combined dataset for model training. Batch effects across datasets were corrected using the “ComBat” function in the “sva”, and PCA confirmed that inter-dataset variations were effectively minimized. The normalized and batch-corrected expression matrix was then randomly split into a training set (70%) and a validation set (30%) to construct supervised classification models distinguishing OA from control samples.

Four supervised machine-learning algorithms were implemented to capture diverse decision boundaries: (1) XGBoost (nrounds = 500, max_depth = 3, eta = 0.1, gamma = 0, colsample_bytree = 0.8, min_child_weight = 1, subsample = 0.5); (2) RF (ntree = 500, mtry = 4–60 by 1); (3) SVM with a radial basis kernel (C = [0.1, 0.5, 1, 2, 5, 10], sigma = [0.01, 0.05, 0.1, 0.5, 1, 5]); and (4) GBM (n.trees = 500, interaction.depth = 3, shrinkage = 0.1, n.minobsinnode = 10). Hyperparameter optimization for each model was conducted through repeated fivefold cross-validation (CV), repeated 10 times using different random seeds (set.seed(1:10)) to ensure stability and reproducibility.

Model performance was primarily assessed using the area under the precision–recall curve (AUCPR). Receiver operating characteristic (ROC) curves and corresponding AUC values were also reported for cross-validation. Feature importance was computed via the “varImp()” function in caret, which measures each gene’s contribution to predictive performance based on model-specific principles: XGBoost – information gain across tree splits; RF – mean decrease in Gini impurity; SVM – permutation-based sensitivity analysis; GBM – cumulative relative influence during boosting iterations. Top-ranked genes were visualized through variable-importance plots, and their intersections were analyzed using UpSet plots to identify genes recognized across multiple algorithms.

### Drug–target interaction prediction and molecular docking

To explore the therapeutic potential of the key macrophage-associated genes RHOB and PDK4, we first performed drug–gene interaction analysis using the Drug–Gene Interaction Database (DGIdb, https://www.dgidb.org). Querying DGIdb enabled identification of approved or investigational compounds known or predicted to interact with each gene. The resulting interaction pairs were imported into Cytoscape (v3.10.0) to construct a gene–drug interaction network for visualization and downstream interpretation.

Molecular docking was then carried out to evaluate the binding affinity between candidate compounds and their corresponding protein targets. Three-dimensional ligand structures were obtained from the PubChem database, and high-resolution X-ray crystal structures of RHOB and PDK4 were downloaded from the Protein Data Bank (PDB). Blind docking was performed using the CB-Dock2 platform, an enhanced cavity-detection and docking workflow that automatically identifies optimal binding pockets and executes AutoDock Vina for pose prediction and affinity scoring. Docking was conducted with default Vina parameters, and the top-ranked pose within each predicted cavity was retained for analysis. Binding affinity was quantified using the Vina score (kcal/mol), where values > −4 kcal/mol indicate weak or no binding, −7 to −4 kcal/mol indicate moderate binding, and < −7 kcal/mol indicate strong binding. Docking poses were visually inspected using the integrated CB-Dock2 viewer.

### Pseudotime trajectory and cell–cell communication analyses

To investigate the dynamic transcriptional states of the 3 immune cell types in OA synovium, pseudotime analysis was performed using “Monocle2”. The analysis was applied to macrophages, mast cells, and dendritic cells from dataset GSE152805, and the resulting trajectories were visualized in two dimensions. Root and terminal states were assigned based on marker-gene expression patterns and trajectory topology. Genes differentially expressed along pseudotime were identified, and GO enrichment analysis was used to characterize stage-specific biological processes.

To assess intercellular communication among these immune subsets, we used the “LIANA” R package (rank_aggregate function), which integrates results from 6 complementary ligand–receptor inference methods: CellPhoneDB, Connectome, log2FC, NATMI, SingleCellSignalR, and CellChat. By aggregating ranked outputs, “LIANA” identifies high-confidence signaling interactions. Networks involving macrophages, mast cells, and dendritic cells were visualized to reveal key communication axes shaping immune responses in OA synovium.

### Statistical analysis

All data processing and analyses in this study were performed with the use of R software. Continuous variables are presented as means ± SD. The Wilcoxon Rank Sum Test was used for comparison between the 2 groups. The results were calculated as correlation coefficients between different molecules by Spearman correlation analysis, and all results were considered significantly different as p<0.05.

## Results

### Single-cell atlas of OA synovium and identification of immune populations with senescence-related transcriptional signatures

**Figure 1:**
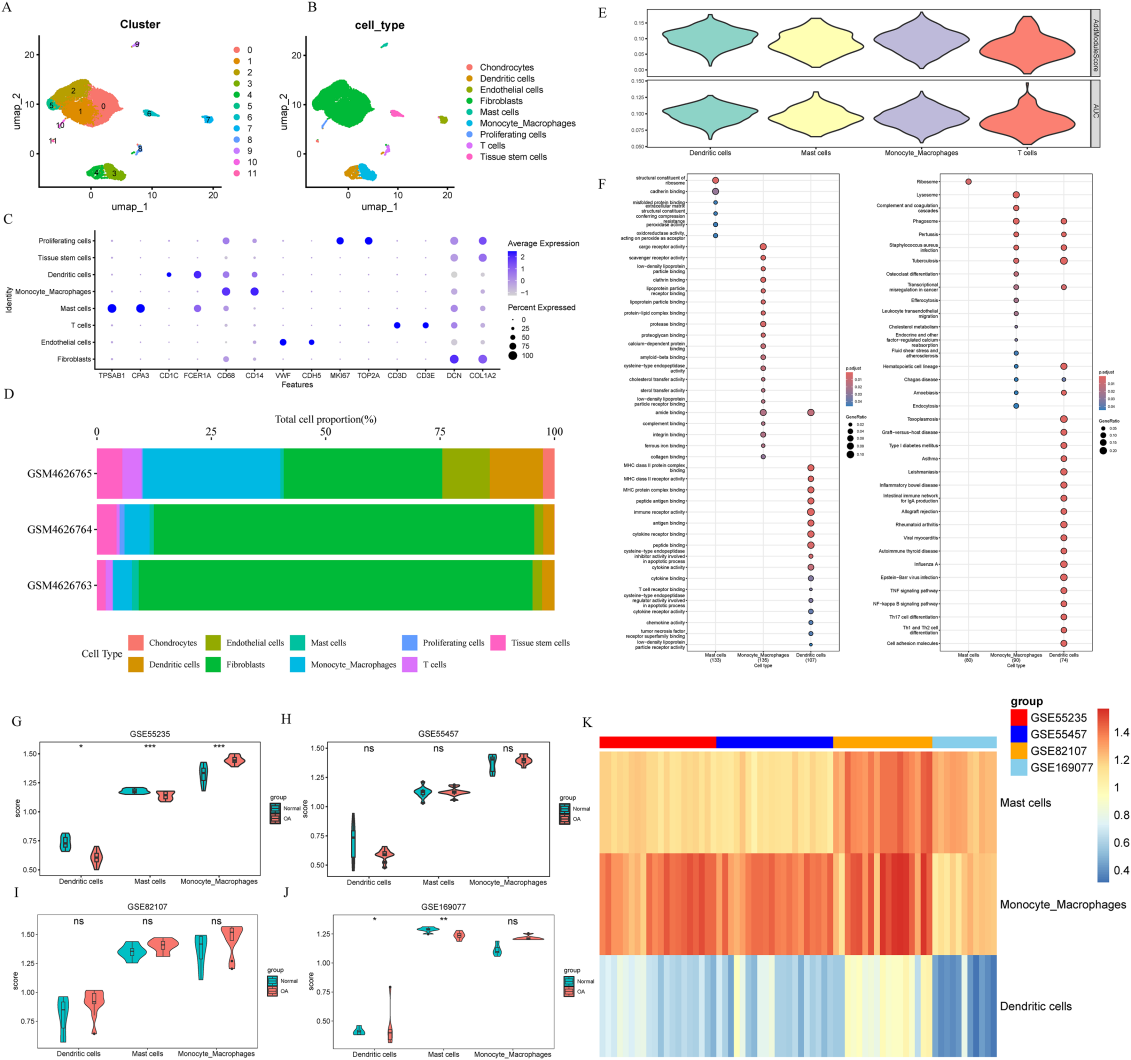
Single-cell atlas of OA synovium and identification of immune populations with senescence-related transcriptional signatures. **(A)** UMAP projection showing 11 transcriptionally distinct clusters in OA synovium. The data are derived from 3 OA synovium and each point represents an individual cell. **(B)** UMAP visualization annotated into 9 major cell types in OA synovium. **(C)** Expression of representative marker genes across annotated cell types. **(D)** The relative abundance of each cell type in OA synovium samples. **(E)** Senescence scores of immune cell types in OA synovium calculated using AddModuleScore and AUC; monocyte/macrophages, mast cells and dendritic cells display the highest senescence scores. **(F)** Gene Ontology (GO) terms and KEGG pathways enriched in the scDEGs of monocyte/macrophages, mast cells and dendritic cells. **(G–J)** GSVA analyses for monocyte/macrophage, mast-cell and dendritic-cell scDEGs in 4 bulk OA datasets: GSE55235 **(G)**, GSE55457 **(H)**, GSE82107 **(I)** and GSE169077 **(J, K)** Heatmap summarizing GSVA scores for the three immune-cell signatures across all samples in the 4 datasets. ns, p>0.05; *p<0.05; **p<0.01; ***p<0.001.

A total of 8,000+ cells from the OA synovial single-cell dataset GSE152805 were retained after quality control filtering and normalization. Dimensionality reduction with UMAP resolved 11 transcriptionally distinct clusters (**Figure 1A**). Using SingleR-based annotation refined by canonical markers, these clusters were assigned to 9 major cell populations: chondrocytes, dendritic cells, endothelial cells, fibroblasts, mast cells, monocyte/macrophages, proliferating cells, T cells and tissue-resident stem cells (**Figure 1B**). Expression patterns of representative marker genes confirmed the accuracy of these annotations (**Figure 1C**). Comparison of cellular composition across OA samples was presented (**Figure 1D**).

To determine which immune populations exhibited the highest senescence activities, we quantified per-cell enrichment of a 279-gene senescence signature using both “AddModuleScore” and “AUCell”. The two methods yielded consistent results, demonstrating that monocyte/macrophages, mast cells, and dendritic cells carry the highest senescence-associated scores among all annotated cell types (**Figure 1E**).

We next identified scDEGs for these three senescence-enriched populations (**Supplementary Table 1**). To confirm whether the scDEGs identified from monocyte/macrophages, mast cells and dendritic cells reflected their biologically functions, we performed GO and KEGG enrichment analyses. As shown in **Figure 1F**, monocyte/macrophage scDEGs were predominantly enriched in functions related to innate immune activation and phagocytic processes, mast-cell scDEGs were associated with oxidative and stress-response pathways, and dendritic-cell scDEGs were enriched for antigen-presentation and adaptive immune–regulatory functions.

To evaluate the relevance of these immune-cell scDEGs with OA at the tissue level, we applied GSVA to the scDEG signatures of monocyte/macrophages, mast cells, and dendritic cells across 4 independent bulk RNA-seq datasets (GSE55235, GSE55457, GSE82107 and GSE169077). Monocyte/macrophage signatures showed significantly higher GSVA scores in OA compared with control samples in GSE55235 (**Figure 1G**). In the remaining datasets (GSE55457, GSE82107 and GSE169077), monocyte/macrophage scores did not exhibit significant elevations between OA and control groups (**Figures 1H–J**). Mast-cell and dendritic-cell signatures did not exhibit significant elevations between OA and control samples in the 4 datasets (**Figures 1G–J**). An heatmap summarized GSVA scores across all samples in the 4 datasets (**Figure 1K**).

### Machine-learning diagnostic models based on monocyte/macrophage specific scDEGs

**Figure 2:**
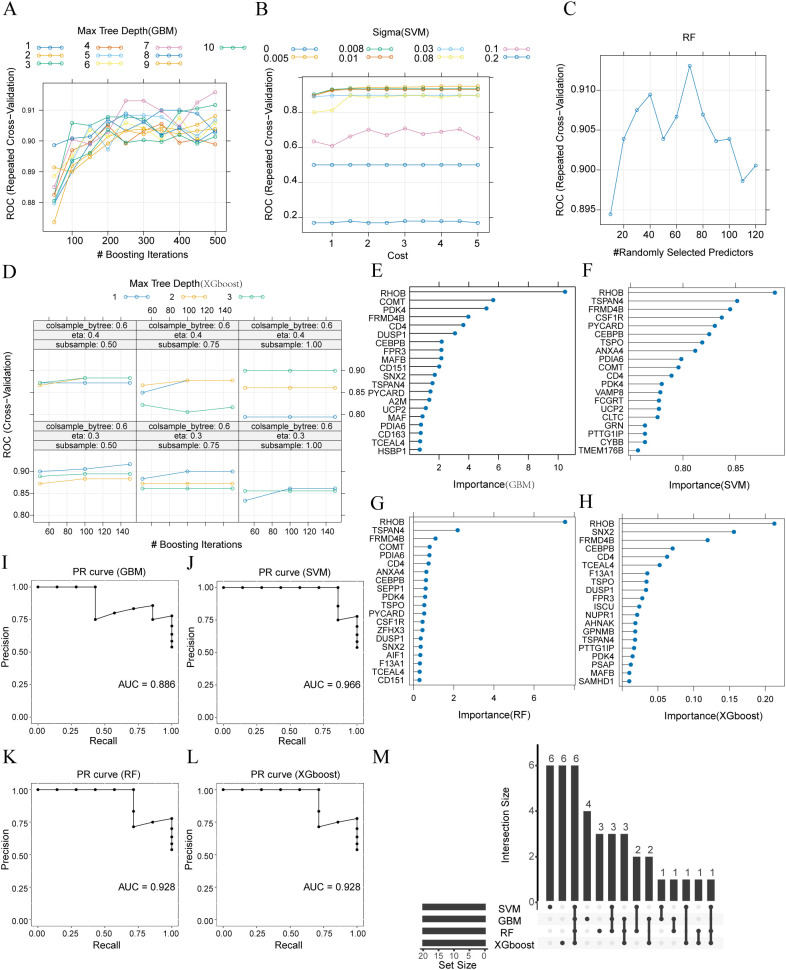
Construction and validation of machine-learning diagnostic models based on monocyte/macrophage-specific scDEGs. **(A–D)** Cross-validation performance surfaces illustrating model accuracy across tested hyperparameter combinations for Gradient Boosting Machine **(A)**, Support Vector Machine **(B)**, Random Forest **(C)** and Extreme Gradient Boosting **(D)**. **(E–H)** Feature-importance plots showing the top-ranked predictive genes identified by each algorithm—GBM **(E)**, SVM **(F)**, RF **(G)**, and XGBoost **(H)**. Importance values represent model-specific metrics: relative influence for GBM, permutation-based sensitivity for SVM, mean decrease in Gini impurity for RF, and gain across tree splits for XGBoost. **(I–L)** Precision–recall curves of the 4 machine-learning models evaluated on the validation set—GBM **(I)**, SVM **(J)**, RF **(K)**, and XGBoost **(L, M)** Upset plot showing the overlap of importance genes across the 4 algorithms (RHOB, TSPAN4, FRMD4B, CEBPB, CD4 and PDK4).

Batch effects across the 4 bulk datasets (GSE55235, GSE55457, GSE82107 and GSE169077) were removed and PCA confirmed that inter-study variation was largely eliminated after correction (**Supplementary Figures 1A, B**). To construct diagnostic models, the combined dataset was randomly split into a 70% training set and a 30% validation set. Hyper-parameter tuning followed the nested 5-fold, 10-repeat cross-validation scheme described above. GBM achieved its optimum with n.trees=500, interaction.depth=7, shrinkage=0.10 and minobsinnode=10, yielding a training AUC of 0.916 (**Figure 2A**). SVM reached maximal performance at σ=0.005 and C = 4 (training AUC = 0.950; **Figure 2B**). RF was optimal with mtry=70 (training AUC = 0.913; **Figure 2C**). XGBoost performed best with nrounds=150, max_depth=2, eta=0.40, gamma=0, colsample_bytree=0.80, min_child_weight=1 and subsample=0.50, giving a training AUC of 0.939 (**Figure 2D**).

The 20 most important genes for each model are illustrated in **Figures 2E–H**. On the validation set, all 4 models retained strong discriminatory power (precision–recall curves, **Figures 2I–L**): GBM AUC = 0.886, SVM AUC = 0.966, RF AUC = 0.928 and XGBoost AUC = 0.928. Intersection of the 4 importance lists identified 6 key genes (RHOB, TSPAN4, FRMD4B, CEBPB, CD4 and PDK4*)*, highlighted in the Upset plot (**Figure 2M**).

### Clinical validation of RHOB and PDK4 expression in synovial macrophages

**Figure 3:**
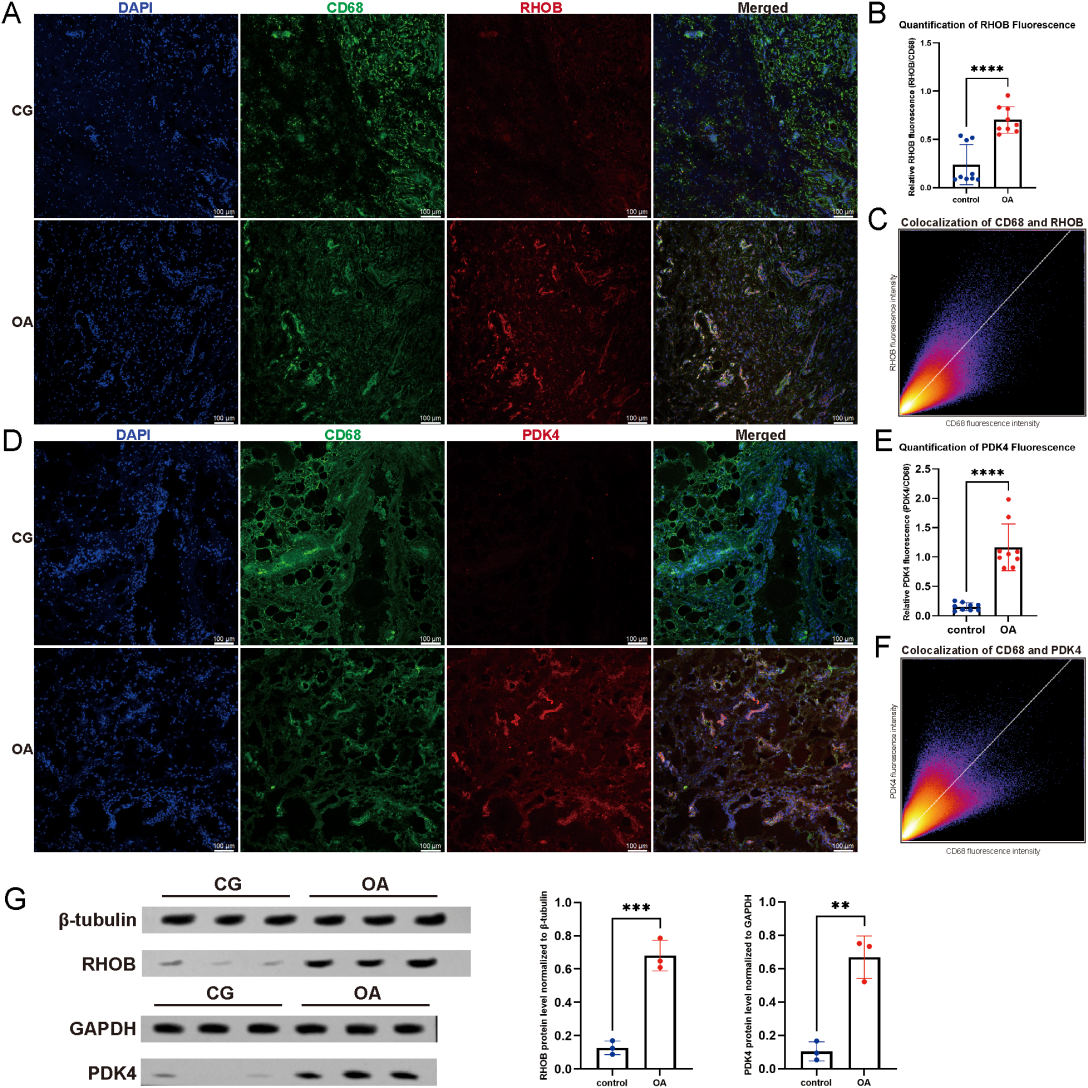
Immunofluorescence and Western blot validation of RHOB and PDK4 expression in synovial macrophages. **(A)** Immunofluorescence images showing RHOB (red) colocalized with CD68 (green) in synovial tissue; nuclei are stained with DAPI (blue). **(B)** Quantification of RHOB/CD68 fluorescence intensity ratio. **(C)** Pixel-wise colocalization scatter plot of CD68 versus RHOB fluorescence signals (Pearson’s R = 0.80). **(D)** Representative immunofluorescence images showing PDK4 (red) colocalized with CD68 (green). **(E)** Quantification of PDK4/CD68 fluorescence intensity ratio. **(F)** Pixel-wise colocalization scatter plot of CD68 versus PDK4 fluorescence signals (Pearson’s R = 0.68). **(G)** Western blot analysis of RHOB and PDK4 protein expression in synovial tissues from OA patients and controls. CG, control group; OA, OA group. **p < 0.01, ***p < 0.001, ****p < 0.0001.

To validate the expressions of the key genes RHOB and PDK4 in OA synovium, immunofluorescence staining and Western blotting were performed on synovial tissues from OA patients and controls. In OA samples, RHOB staining (red) was strongly enriched within CD68^+^ macrophage-rich regions (**Figure 3A**), and quantification of fluorescence intensities demonstrated a significant elevation of the RHOB/CD68 ratio relative to controls (**Figure 3B**; p < 0.0001). Pixel-wise scatter analysis further confirmed robust colocalization between RHOB and CD68, with a Pearson’s correlation coefficient of 0.80, indicating tight spatial coupling of RHOB expression to macrophage distribution (**Figure 3C**). Similarly, PDK4 (red) also showed enhanced accumulation in CD68^+^ areas of OA synovium (**Figure 3D**). Quantitative fluorescence analysis revealed a significantly increased PDK4/CD68 ratio in OA compared with controls (**Figure 3E**; p < 0.0001). Colocalization analysis produced a Pearson’s R of 0.68, indicating substantial overlap between PDK4 expression and macrophage presence (**Figure 3F**). Both RHOB and PDK4 exhibited elevated fluorescence and increased colocalization with CD68^+^ macrophages in OA synovial tissue. Western blotting result showed both RHOB and PDK4 protein levels were markedly increased in OA synovium compared with control tissues, consistent with the immunofluorescence results (**Figure 3G**).

### Drug–target prediction and molecular docking for RHOB and PDK4

**Figure 4:**
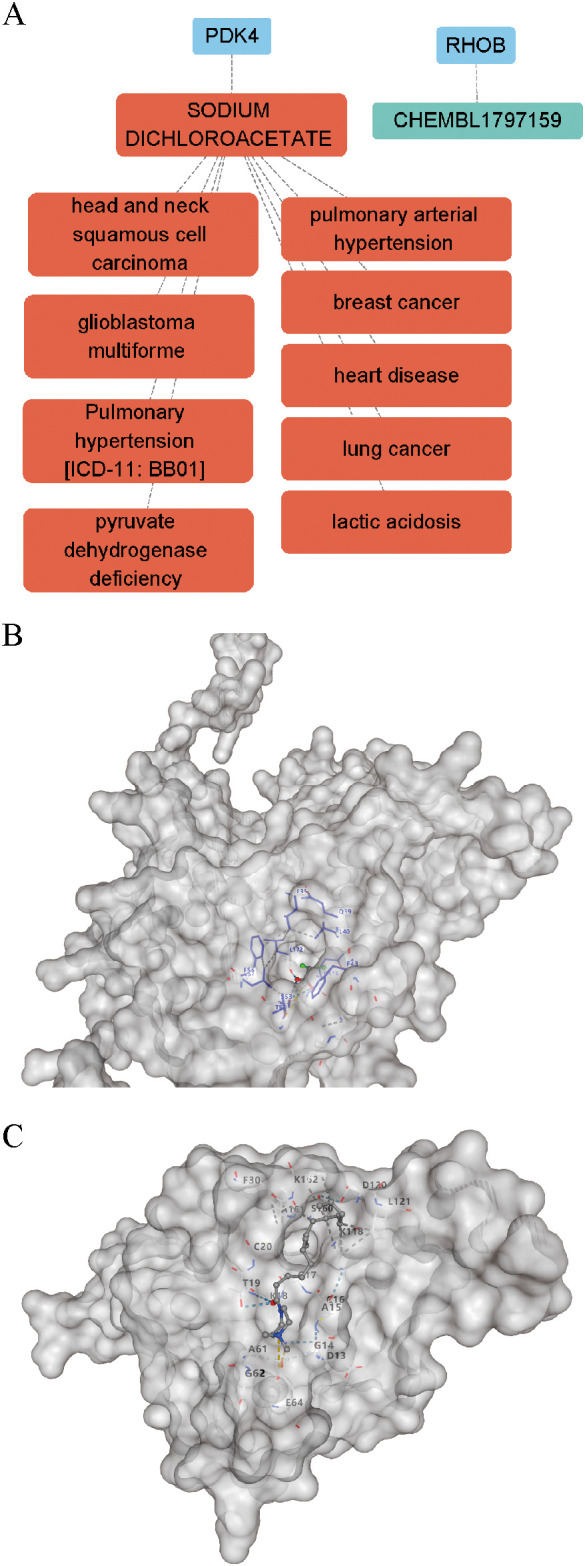
Drug–gene interaction mapping and molecular docking for RHOB and PDK4. **(A)** DGIdb-derived interaction network displaying the selected gene–compound pairs—PDK4 with SODIUM DICHLOROACETATE (DCA) and RHOB with CHEMBL1797159. The network also shows known disease associations of DCA. **(B)** Molecular docking pose of PDK4 with SODIUM DICHLOROACETATE (Vina score = −4.0 kcal/mol). **(C)** Molecular docking pose of RHOB with CHEMBL1797159 (Vina score = −5.2 kcal/mol).

To explore whether RHOB and PDK4 possess tractable therapeutic potential, each gene was queried (**Supplementary Table 3**). Among the predicted interactions, SODIUM DICHLOROACETATE (DCA) was identified as a potential modulator of PDK4, and CHEMBL1797159 was identified as a potential ligand for RHOB. DGIdb annotations revealed that DCA is clinically associated with multiple diseases, including pulmonary hypertension, breast cancer, glioblastoma multiforme, head and neck squamous cell carcinoma, heart disease, lactic acidosis, lung cancer, pulmonary arterial hypertension and pyruvate dehydrogenase deficiency, highlighting its relevance as a repurposable drug (**Figure 4A**).

To evaluate the structural feasibility of these interactions, molecular docking was performed using CB-Dock2. Docking simulations demonstrated moderate predicted affinity between PDK4 and DCA (Vina score = –4.0 kcal/mol; **Figure 4B**), and between RHOB and CHEMBL1797159 (Vina score = –5.2 kcal/mol; **Figure 4C**).

### Machine-learning diagnostic models based on mast cell and dendritic cell specific scDEGs

**Figure 5:**
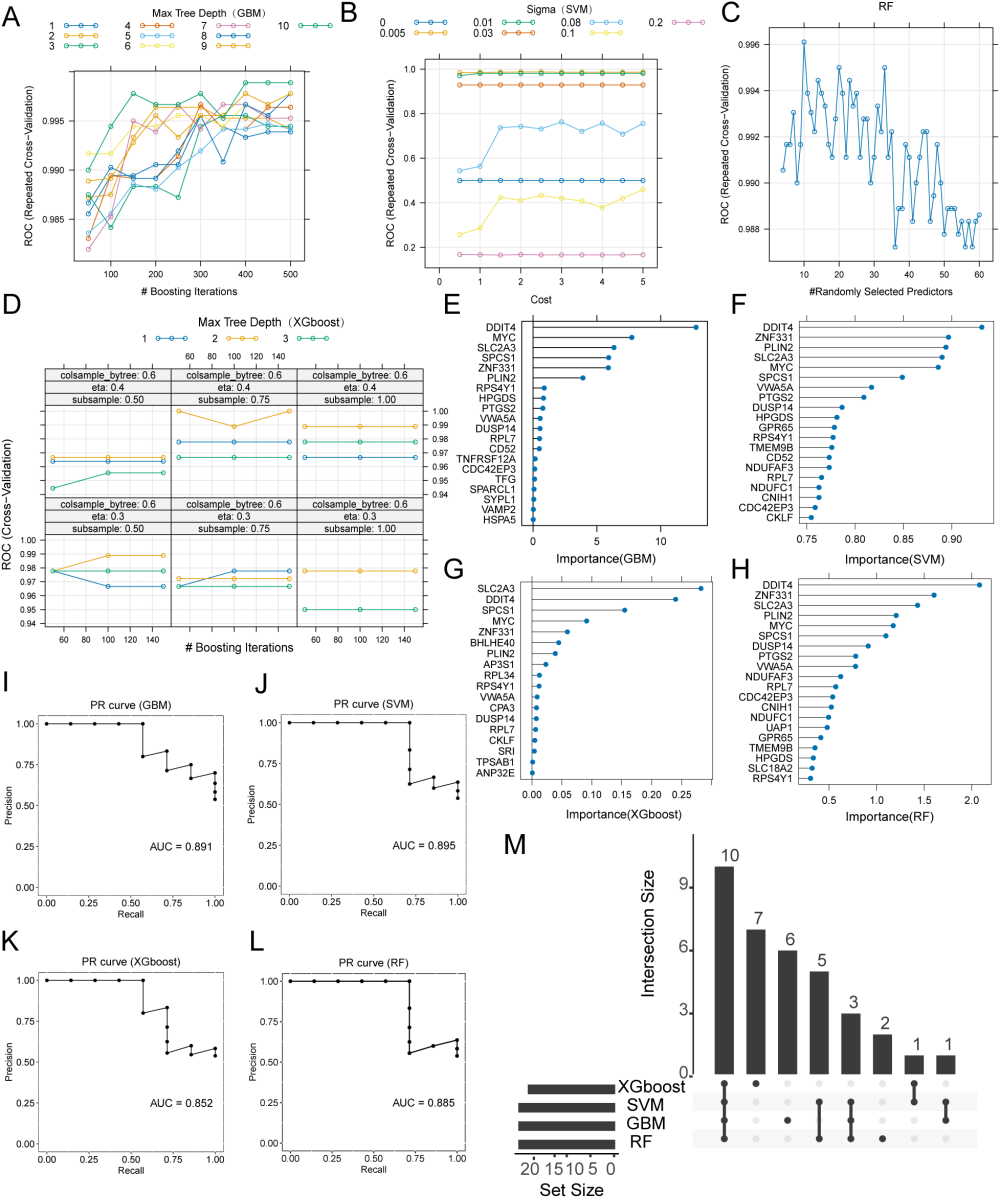
Construction and validation of machine-learning diagnostic models based on mast cell-specific scDEGs. **(A–D)** Cross-validation performance surfaces for GBM, SVM, RF and XGBoost. **(E–H)** Top 20 importance-ranked genes identified by each algorithm. **(I–L)** Precision–recall (PR) curves on the validation set. **(M)** UpSet plot showing the overlap of importance genes across the 4 algorithms (DDIT4, ZNF331, PLIN2, SLC2A3, MYC, SPCS1, VWA5A, DUSP14, RPS4Y1 and RPL7).

**Figure 6:**
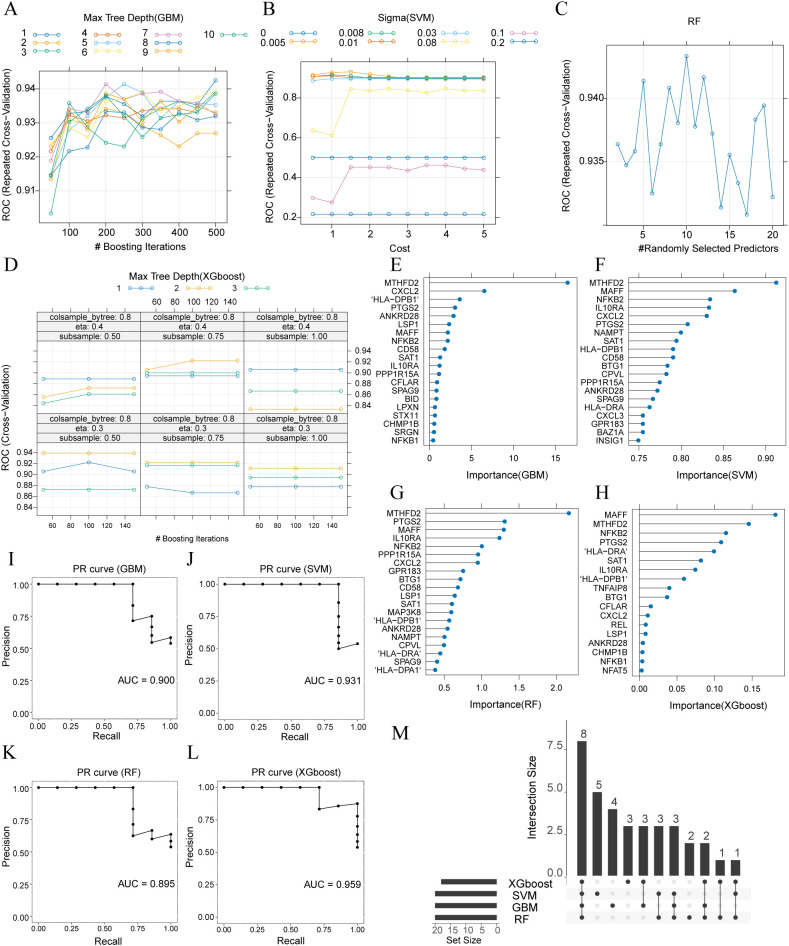
Construction and validation of machine-learning diagnostic models based on dendritic cell-specific scDEGs. **(A–D)** Cross-validation performance surfaces for GBM, SVM, RF and XGBoost. **(E–H)** Top 20 importance-ranked genes identified by each algorithm. **(I–L)** Precision–recall (PR) curves on the validation set. **(M)** UpSet plot showing the overlap of importance genes across the 4 algorithms (MTHFD2, MAFF, NFKB2, IL10RA, CXCL2, PTGS2, SAT1 and ANKRD28).

Using the same modelling framework applied to monocyte/macrophages, we next constructed diagnostic models based on mast cell–specific and dendritic cell–specific scDEGs. All four algorithms (GBM, SVM, RF and XGBoost) were tuned by repeated cross-validation, and their cross-validation performance surfaces are shown in **Figures 5A-D**, **6A–D**. Models of both cell types, demonstrated strong internal performance, with training AUCs ranging from 0.93 to 1.00. Feature-importance analyses identified distinct sets of predictive genes for each algorithm (**Figures 5E–H**, **6E–H**). When evaluated on the validation set, mast-cell–based models achieved AUCPR values between 0.852 and 0.895 (**Figures 5I–L**), while dendritic-cell–based models showed AUCPR values between 0.895 and 0.959 (**Figures 6I–L**). Intersection analyses revealed 10 key mast-cell genes (DDIT4, ZNF331, PLIN2, SLC2A3, MYC, SPCS1, VWA5A, DUSP14, RPS4Y1 and RPL7; **Figure 5M**) and 8 key dendritic-cell genes (MTHFD2, MAFF, NFKB2, IL10RA, CXCL2, PTGS2, SAT1 and ANKRD28; **Figure 6M**).

### Pseudotime trajectory analysis and cell–cell communication patterns

**Figure 7:**
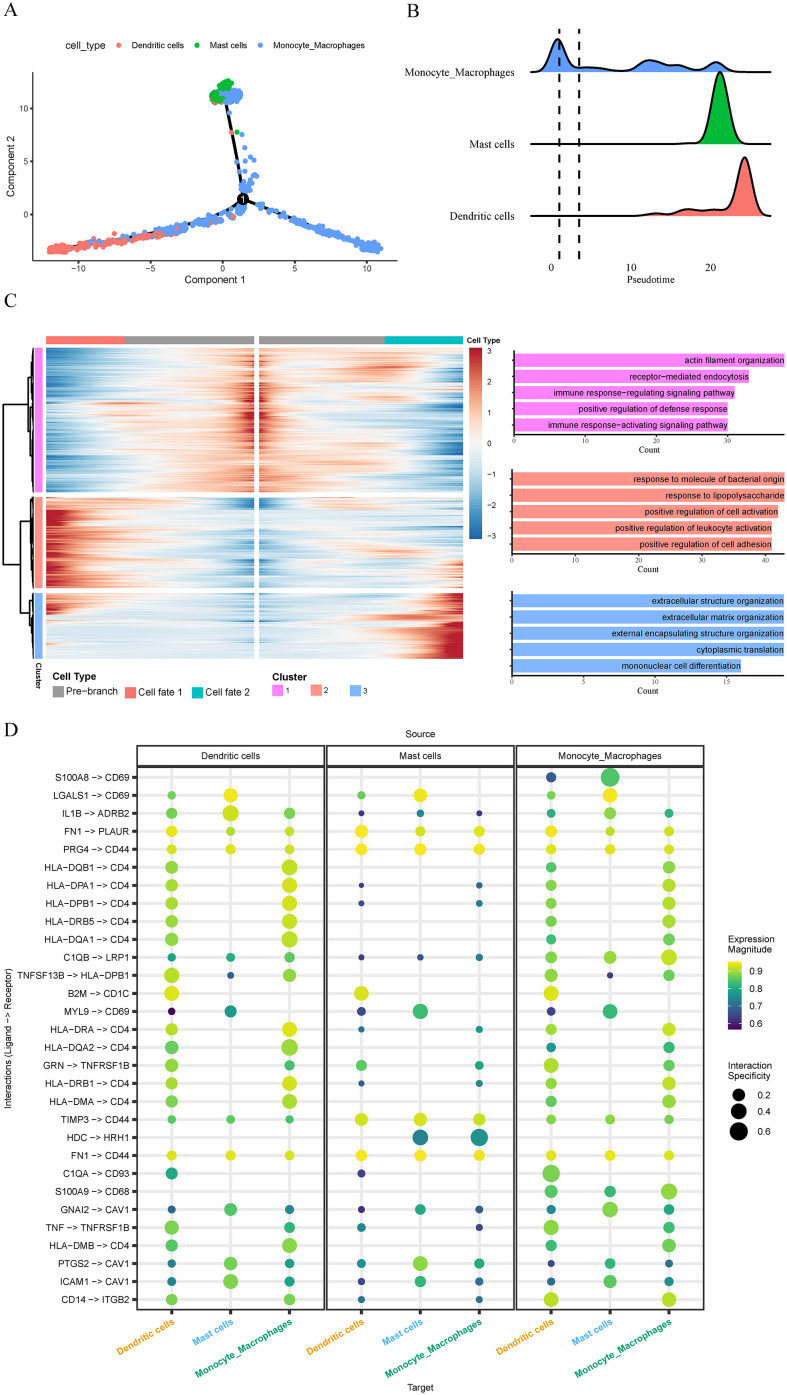
Pseudotime trajectories and intercellular communication among macrophages, mast cells, and dendritic cells in OA synovium. **(A)** Two-dimensional projection colored by pseudotime-defined clusters; arrows indicate the inferred progression direction (right→left). **(B)** Cell-density overlay showing the distribution of macrophages (root), mast cells (intermediate), and dendritic cells (terminal). **(C)** Heat-map of differentially expressed genes across the three pseudotime clusters with representative Gene Ontology terms illustrating early (cluster 2, orange), intermediate (cluster 1, purple), and late (cluster 3, blue) transcriptional programs. **(D)** Ligand–receptor interaction network with dot size indicating confidence and color intensity representing interaction strength, summarizing communication patterns among macrophages, mast cells, and dendritic cells.

Pseudotime trajectory analysis revealed a continuous transcriptional progression connecting the 3 immune populations. Along the inferred trajectory, macrophages were enriched toward the early pseudotime region, where they exhibited the highest transcriptional activity. However, macrophages also extended along the trajectory into intermediate and late pseudotime. Mast cells occupied an intermediate pseudotime zone and dendritic cells were positioned predominantly at late pseudotime (**Figures 7A, B**). Genes varying significantly along pseudotime were grouped into three major expression modules (**Figure 7C**). The early module (orange) showed high expression at the beginning of the trajectory and was associated with inflammatory stimulus sensing and leukocyte activation. The intermediate module (purple) peaked in the mid-trajectory and contained genes linked to cytoskeletal remodeling, endocytic activity, and immune regulatory processes. The late module (blue) increased toward the trajectory terminus and was enriched for extracellular-matrix organization and mononuclear cell differentiation.

Cell–cell communication analysis identified several ligand–receptor interactions among the three immune subsets (**Figure 7D**). Macrophages displayed the largest number of high-confidence interactions, including S100A8-CD69, TNF–TNFRSF1B, HLA class II–CD4, C1QB–LRP1, and PRG4–CD44, suggesting their central role in coordinating inflammatory and regulatory signaling. Dendritic cells showed strong antigen-presentation–related interactions, whereas mast cells exhibited more restricted communication centered on adhesion and inflammatory mediator pathways. These results collectively reveal a structured signaling network linking the three immune cell populations in the OA synovial microenvironment.

## Discussion

In this study, we identified macrophages, mast cells, and dendritic cells as the immune populations with senescence-related transcriptional signatures in OA synovium and defined their transcriptional signatures by identifying subset-specific scDEGs through single-cell analysis. Multi-algorithm machine-learning models highlighted RHOB and PDK4 as robust macrophage-associated biomarkers, which were further validated in OA synovial tissues and associated with potential therapeutic compounds identified through drug–gene interaction and molecular docking. Pseudotime and cell–cell communication analyses further indicated that macrophages initiate and centrally coordinate the synovial inflammatory response, whereas mast cells and dendritic cells contribute distinct mid- and late-stage functions within the synovium immune microenvironment.

Although macrophages are widely recognized as key regulators of synovial inflammation and joint degeneration, accumulating evidence suggests that immune dysfunction in aging extends beyond macrophages and contributes to a chronic pro-inflammatory microenvironment (27, 28). Our study identified macrophages, mast cells, and dendritic cells as the prominent senescent immune subsets in the synovium, which expands the current understanding of immune dysregulation in OA synovium. Functional enrichment analysis further confirmed that the scDEGs identified from macrophages, mast cells, and dendritic cells were highly consistent with the established biological roles of these immune subsets, indicating that these immune gene signatures captured by our analysis robustly reflect their underlying cellular functions. Previous study have proposed that crosstalk between joint cells and tissues play essential roles in OA but specific mechanism remains unclear (29). Pseudotime reconstruction revealed a coordinated inflammatory continuum in which macrophages, mast cells, and dendritic cells assume temporally ordered yet functionally interconnected roles during OA synovitis. Macrophages dominated early pseudotime, expressing stimulus-sensing and leukocyte-activation programs that are characteristic of inflammatory initiation. Importantly, macrophages did not disappear after the early phase; instead, their transcriptional activity extended into intermediate and late pseudotime, consistent with their recognized role as sustained regulators of joint inflammation and tissue remodeling (30). This temporal prominence was mirrored in the communication network: macrophages engaged in extensive ligand–receptor signaling with both mast cells and dendritic cells, positioning them as upstream controllers of synovial immune activation. Mast cells emerged in intermediate pseudotime, where enrichment of oxidative-stress, cytoskeletal-remodeling, and endocytic pathways suggests that they respond to macrophage-derived inflammatory cues and amplify the evolving synovial microenvironment. Their position implies a “response-and-amplification” function—bridging early macrophage activation with downstream immune escalation. Dendritic cells accumulated in the terminal pseudotime region, aligning with late-stage functions including extracellular matrix and extracellular structure organization, indicating that they may participate in late-stage tissue remodeling in OA. A macrophage-derived S100A8–CD69 interaction represented a mast-cell–specific axis. Given the DAMP properties of S100A8/A9 and the pro–inflammatory signaling driven by CD69 (31, 32), this pathway likely primes mast cells and reinforces their intermediate-stage “inflammatory amplification” role. Mast cells at mid-pseudotime exhibited oxidative-stress, cytoskeletal-remodeling, and endocytic programs, aligning with their capacity to intensify macrophage-initiated inflammation. Dendritic cells occupied terminal pseudotime states, expressing extracellular matrix and extracellular structure organization programs. Their maturation may be driven by macrophage-derived TNF–TNFRSF1B, a key pathway promoting synovial fibroblast activation and joint destruction (33), thereby supporting chronic synovitis and late-stage tissue remodeling. Together, these findings outline a macrophage-centered regulatory cascade in which macrophages initiate inflammation, activate mast cells to amplify mid-stage responses, and subsequently drive dendritic-cell maturation to sustain late-stage remodeling.

By integrating scRNA-seq–derived immune-cell–specific signatures into 4 complementary machine-learning algorithms, this study achieved a cross-validated strategy for biomarker discovery. The use of multiple algorithms, each relying on distinct principles of feature selection, substantially reduces the instability and model-specific bias inherent to single-method analyses. Moreover, because the input features were derived from immune-cell–specific signatures defined at single-cell resolution, they more accurately capture the functional states of senescent immune subsets within the OA synovium. This cellular specificity increases the likelihood that the selected markers represent true disease-driving pathways and therefore yields more biologically meaningful and actionable therapeutic targets. Six macrophage-associated genes (RHOB, TSPAN4, FRMD4B, CEBPB, CD4, and PDK4), 10 mast cell-associated genes (DDIT4, ZNF331, PLIN2, SLC2A3, MYC, SPCS1, VWA5A, DUSP14, RPS4Y1, and RPL7), and 8 dendritic cell-associated genes (MTHFD2, MAFF, NFKB2, IL10RA, CXCL2, PTGS2, SAT1, and ANKRD28) were identified.

Among these gene sets, RHOB and PDK4 were prioritized for deeper validation because RHOB consistently ranked as the top feature across all 4 machine-learning algorithms, and PDK4 has been repeatedly implicated in senescence-related metabolic regulation, making both genes strong candidates for experimental confirmation and therapeutic exploration (34, 35). Immunofluorescence analyses confirmed that both genes exhibit marked upregulation and strong spatial colocalization with macrophages in OA synovium, reinforcing their pathogenic relevance. RHOB, a member of the Rho GTPase family, is known to regulate intracellular signaling through interactions with EGFR, RAS, and PI3K–Akt–mTOR pathways, as well as cytoskeletal organization and vesicular trafficking (36–40). Although RHOB is often studied in tumorigenesis and aging, accumulating evidence links RHOB dysregulation to inflammatory responses. Importantly, genetic association studies have repeatedly implicated RHOB in OA susceptibility, although mechanisms have remained unclear (41, 42). Our finding suggests that elevated RHOB in OA synovial macrophages may contribute to a senescence-associated inflammatory phenotype, enhancing SASP-like cytokine release and joint inflammation. PDK4 (pyruvate dehydrogenase kinase 4), a mitochondrial serine/threonine kinase that phosphorylates and inhibits the pyruvate dehydrogenase complex, promotes a metabolic shift from oxidative phosphorylation to aerobic glycolysis, leading to lactate accumulation, mitochondrial dysfunction, and reactive oxygen species generation (34, 43–45). Recent studies indicate that PDK4 functions as a context-dependent metabolic regulator rather than a uniformly deleterious OA gene. In chondrocytes, PDK4 is downregulated in OA and exerts protective effects through activation of the PPAR pathway, reducing inflammatory cytokines and matrix-degrading enzymes (46). In contrast, multi-omics analyses have identified PDK4 as an OA-associated mitochondrial hub gene predominantly associated with macrophages (47). Our data indicate that PDK4 was a key regulator of OA synovium macrophages. Within macrophages, PDK4 likely contributes to senescence and inflammatory metabolic reprogramming. These findings support a cell type–specific model in which PDK4 is protective in chondrocytes but potentially pathogenic in synovial macrophages. This duality raises important therapeutic considerations, as systemic PDK4 inhibition may suppress macrophage-driven inflammation while risking disruption of chondrocyte-protective PPAR signaling. The druggability analyses further highlight their translational potential. CHEMBL1797159, predicted to bind RHOB, represents a previously unexplored small-molecule candidate with plausible structural affinity supported by molecular docking. Sodium dichloroacetate (DCA), a clinically used metabolic modulator, emerges as an attractive repurposing candidate for PDK4. While docking results indicate feasible binding interactions, functional assays will be essential to establish causal therapeutic relevance.

This study has several limitations that should be acknowledged and addressed in future research. First, the sample size of the datasets used in this study was limited. The small sample size may restrict the statistical power of the analysis and thus affect the generalizability and robustness of the study findings. Second, due to the small number of synovial specimens used for experimental validation, a rigorous power analysis could not be performed in advance to determine the optimal sample size. Third, this study did not set up a control group for senescence. Instead, we used 2 senescence gene set scoring methods (AddModuleScore and AUCell) to evaluate cellular senescence. This indirect evaluation method may not fully reflect the actual senescence status of cells, and the lack of a direct senescence control group may lead to potential biases in the interpretation of the results. Fourth, the senescence gene list used in this study was derived from cancer senescence-related transcriptomic signatures. This study only represents a preliminary exploration of the senescence characteristics of synovial immune cells in OA.

In summary, we identified macrophages, mast cells, and dendritic cells as the key immune subsets with senescence-related transcriptional signatures in OA synovium. These cells form a coordinated inflammatory continuum, with macrophages initiating and sustaining synovial activation, mast cells amplifying mid-stage responses, and dendritic cells driving late-stage remodeling. RHOB and PDK4 emerged as robust macrophage-associated markers with potential druggable relevance, highlighting macrophage-centered senescence pathways as promising therapeutic targets in OA.

## Data Availability

The original contributions presented in the study are included in the article/**Supplementary Material**. Further inquiries can be directed to the corresponding author.
