# Supplementary figures and images for: Immune cells with senescence-related transcriptional signatures orchestrate the inflammatory continuum in osteoarthritis synovium: a single-cell and machine learning study
Source: Front Immunol. 2026 Apr 17;17:1774722. doi: 10.3389/fimmu.2026.1774722 (PMC13132789; doi:10.3389/fimmu.2026.1774722)

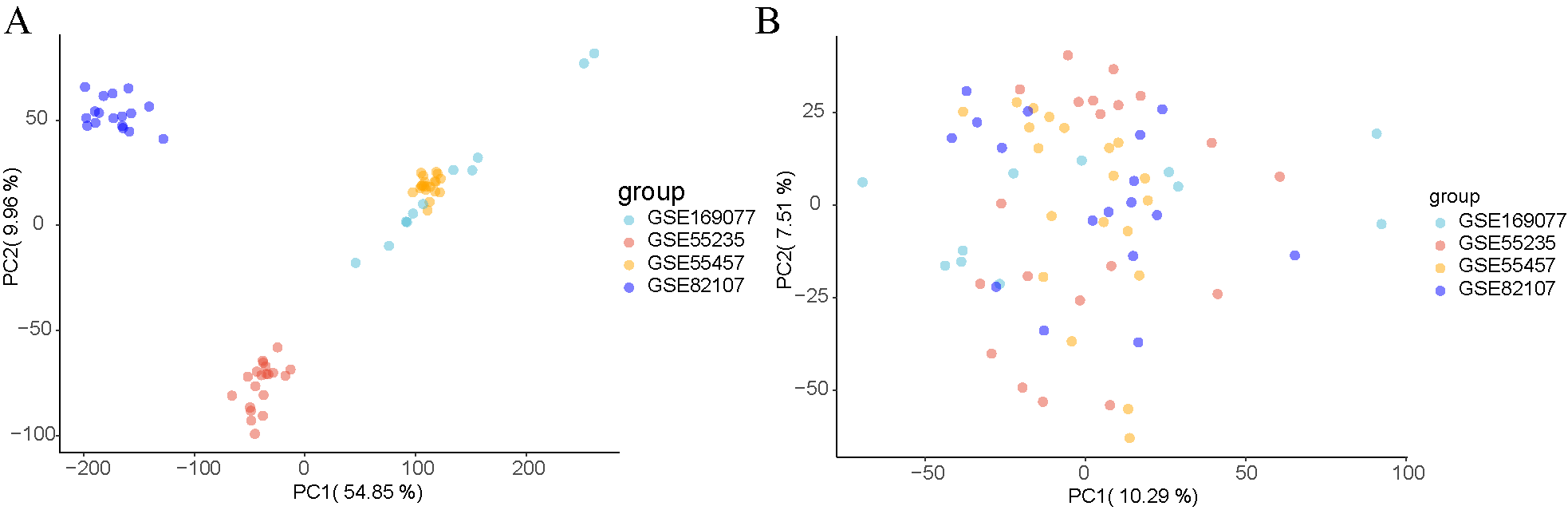

Supplement: Supplementary file 5 [file Image1.tif]
